# Mr. Oliphant's Cases

**Published:** 1800-12

**Authors:** Isaac Oliphant

**Affiliations:** Percy Street


### *To the Editors of the Medical and Phyſical Journal.*

Gentlemen,

Be pleaſed to inſert, if you think proper, the following Caſes in your valuable periodical Journal. I am,

Your humble ſervant,

*Percy Street, Nov.* 21, 1800. ISAAC OLIPHANT.

---

Mrs. Oſborn, now aged 57, wife of Mr. Oſborn, breeches-maker, South-ſtreet, Mancheſter ſquare, nineteen years ago received a puſh on her right breaſt, and ſoon after a violent preſſure from an iron rail. Conſiderable pain was felt from the laſt, and a hardneſs left, with tenderneſs to the touch. In ſome ſhort time, there aroſe a puckering of the ſkin all around around the hardnefs; in procefs the furrows became exceffive, all concentric to the induration, and compared by her to *binding strings.*

Inflammation fupervened, all the breaft appeared comprized in it; fuppuration came on, and feparated the induration from its furrounding attachment to the breaft. This procefs was accompanied with cold fhivering, ficknefs, and vomiting, as happens ordinarily in fuppuration and extenfive floughings. When the feparation was complete, the indurated portion came out, according to her term, like a cork, leaving a gaping hole.

After the expulfion of the difeafed portion, the puckering fkin unfolded to pafs into the cavity; the fkin crept in, becomcoming inverted. The ulcerated furface was very little fenfible, but the breaft was very fufceptible and tender. From the cavity, exceffive hæmorrhage enfued, happening particularly in the night, fo as to deluge the bed-cloaths, and reduced her to extreme weaknefs.

During the ulcerating procefs a cup full of cliver-juice was drank every morning, when it could be had; and malt infufion in the different ftages of fermentation with yeaft, made in a jar, to her victuals, and to an unlimited quantity.

The glands in the axilla were greatly enlarged during the procefs of induration and feparation; and the band of communicating lymphatics was very fenfibly increafed. The arm was fwelled, of a cumbrous weight, and rendered almoft entirely ufelefs.

Seven years ago I faw her; the communicating lymphatics between the breaft and axilla were alone in a condition of ulcer; the breaft was entirely abforbed, and gone, but axillary fwelling continued confiderable.

She had for fome time ufed an ointment of oil, wax, and rofin, (the ointment in common ufe with the pretenders to cure cancers) which I wifhed her to continue, as well as her other plan. The fwelling of the arm and in the axilla gradually leffened and difappeared, and the fore by degrees healed, and has been well for five years paft.

There was fome reafon to believe, that the change which took place in her breaft was owing to fupervening febrile affection occurring two or three times in the autumn, attended with cholera morbus to a dangerous degree; her breaft was in its worft ftate at this time; and fince its cure, fhe has been free from fuch attacks.

Mrs. Craib, of Tottenham-court-road, aged 46, has had three children, the laft fifteen years ago; was never able to fuckle with the left breaft, but had no great inconvenience from this circumftance. The menfes hitherto regular, till pleurify feized feized her; otherwife fhe was extremely healthy, and conftantly employed in an active life.

Two years ago fhe got a violent bruife on the upper part of her left breaft; the effects fubfided as well as they ufually do in common cafes, only there was left fome hardnefs with a little occafional pain.

Six months after fhe received another bruife on the fame part, and all the confequences of contufion, fwelling, livid colour, and pain, were greater than in the former accident; and the remaining pain was more fevere, and the hardnefs of greater extent. In the recent ftate, repeated applications of leeches were ordered; and when the active condition of the difeafe was fuppofed to be removed, fome faponaceous camphorated embrocation was applied.

At this time the hardnefs was detached, and moveable from the general glandular ftructure of the breaft, when fhe went under the care of a friend, not of the profeffion, but who was a clofe reader of medical books; he had culled from thence active applications, but of courfe applied them at random. The intention was to procure fuppuration in the hardnefs, after means of difcuffion had failed; and one application was a poultice of pigeon's dung, that produced exceffive burning and moft excruciating pain; which, however, I believe, changed totally the difeafed condition, but increafed its extent. It now reached over much more of the breaft, and made the whole a confolidated mafs of difeafe, and attended with an extended irritation to the axilla and other breaft, wherein enlargements arofe.

The next perfon under whofe care Mrs. Craib fell, was a man who kept a chandler's fhop in her neighbourhood, and had been a porter to a furgeon. He had the boldnefs to pronounce his new patient curable in fix weeks, and that he would fet about it. He began his courfe by fprinkling it with a white powder, fuppofed burnt allum, which excited much pain, and covered the whole with a favourite ointment that he always carried away with him. This would not do, but he faid, it was only preparatory to ripen for the knife.

Mrs. Craib now got a recommendation to an eminent and experienced furgeon, who directly faw the marks of bad treatment in her breaft, and expreffed aftonifhment, hearing her cafe, that fhe had not had regular affiftance, and offered his fervices of recommendation to a public inftitution, which, however, fhe declined accepting.

She was depreffed with the profpect before her, and tormented with fhooting throbbing pains, particularly at the upper part of the breaft, attended with an oozing, irritating fanies, that excoriated as it extended. There was an ulceration below the nipple, nipple, about the fize of a fhilling, and one above of about half the fize, with jagged edges, covered at the bottom with a tomentous flough, of a yellowifh colour. The fkin furrounding the ulcers was level with the reft of the breaft.

In addition to the above, when fhe firft applied to me, the whole breaft was a dark red confolidated tenfe mafs, much like a large carbuncle, firmly fixed to the cheft, and fhe had a fwelling in the arm-pit the fize of a pullet's egg; chronic inflammation had feized feveral cuticular glands around, particularly between the breafts, and moft in the upper part. There was an adventitious hardnefs in the other, which fhe faid, arofe from the painful applications to the ulcerated one: I dare fay it would have weighed three-fourths of a pound.

In fome places there were interftitial fuppurations; a round hole with matter on the furface that could not be wiped away, refembling fea-fcurvy, as happens in habits who have ulcers, particularly in the legs; there are often fuch ulcerations of the fkin, at different diftances.

Her hands were affected with flight rhagades, and her left arm was confiderably fwelled; her pulfe was quick, but, I believe, not too frequent; her health conftitutionally unimpaired, and perfectly regular. She flept well without an anodyne; and, in fhort, had nothing to complain of but her ulcerated breaft, and fwelled weak arm, never feeling any affection from the other breaft.

She put herfelf under my care the 8th of June laft, and, to allay the irritation arifing from improper applications and want of dreffing, a fedative opiate folution was applied to the ulcerations, and I covered the whole breaft with foap cerate: She was directed to take a dofe, and repeat it occafionally, of magnefia vitriolata, and daily, three times a day, four ounces of the aqua mephitica alkalina, and to bleed the moft tenfe part with leeches. This had the defired effect in removing the fuperinduced irritation; and, on a fubfidence of the fwelling, there was a floughing, and ftench from the fore: but I was obliged to change this application for the fermenting poultice, and this application was continued to the 18th, with the intended effect. The fore was as eafy as fuch a difeafed furface almoft in any cafe could be fuppofed to be. The tendernefs in the axilla was reduced; from not being able to bring her arm to her fide, for fome time paft, nor lay on that fide, fhe could nearly do both; the choppings were much leffened, and fhe felt herfelf able to attend conftantly her fhop, and had good nights reft. A favourable circumftance alfo was, that no bleeding attended on this ulceration; as the whole breaft, and its veffels, appeared enveloped in fuch difeafe, that I fhould have had little reafon to to expect their contracting to reftrain hæmorrhage if it had occurred.

Having received a circular letter, in common with others in the profeffion, from Dr. Nifbet, fignifying he intended to confine his practice to the treatment of Cancer, Scrophula, and Phthifis, I had the confent of Mr. Craib to have his advice, on the 18th of June, and he prefcribed a grain of opium every night, a common drink of a fmall portion of fixed vegetable alkali and fulphur, diffufed in water, and a fulphurated opening electuary; the breaft to be dreffed with fimple cerate; and he faid that it was a hopelefs cafe, and that nothing could be done but palliation.

However, he wifhed to give her an antimonial alterative pill, of his own preparation, which I admitted of, wifhing to wave delicacy for the poffible benefit of my patient; but fhe only loft ftrength. Hopes of fuppuration in the principal part of the difeafe were entertained, but it did not happen.

She foon was obliged to keep her bed-room, and fpent much of her time in bed; indeed, working in her fhop leffened the profpect of advantage from any treatment.

July 18th, in the evening, fhe was taken with a fevere rigor, and I gave an opiate, and a dofe of ipecac. in aq. ammon. acet. which brought on a profufe fweat; fhe had three fuch rigors in twenty days, which appeared to be owing to the fedative action of new-formed floughs, and which, at this time, exhaled fuch a fœtor that Mr. Craib was obliged to part beds.

There was an ointment rubbed in, into the fide of the ribs, fimilar to the pill, fubftituting fulphur for antimony, alfo of Dr. Nifbet's preparing. The applications were of my fuggefting, and thofe I conceived the leaft fufpicious of irritating, and divefted of unctuofity, which quality is ever hurtful to fuch ulcers.

I was now forry that the plan had been purfued fo long, as no advantage had been got by it, either in the difeafed appearance of the breaft, or the fores; the laft were greatly enlarged, and the breaft had leffened in fize in an equal proportion only to the general wafte in the body. She was now preffed to go into the country as the only refource, but as her journey was to be to Nottingham, fhe wifhed for ftrength to bear fuch fatigue.

On the 16th of Auguft, in the evening, fhe got out of bed to have it put to rights; thinking fhe would fhortly return to it, fhe did not put on her ftays; and in this ftate remained longer up than fhe at firft intended, or thought herfelf able.

For want of her ftays, as fhe thought, and in my opinion rightly, a pain feized her under the right breaft, which fhe apprehended arofe from flatulency, and drank fome glaffes of Madeira wine to difpel it; however, the pain increafed, and I was called at five in the morning, when her pulfe could be hardly felt; fhe was obliged to fit up, was crying out continually with pain, at the fame time preffing forcibly on her fide for eafe.

I bled her to nine or ten ounces, and applied aq. ammon. pur. with a little oil, to excite a fudden irritation, and fave farther weakening by lofing of more blood, and gave a dofe of opium and ipecacuanha directly after.

However, the pain recurring from a fit of coughing, I was obliged to repeat the bleeding about four hours after the firft, to about the fame quantity, both of which were highly buffed and cupped. Her pulfe rofe, and I continued the opiate relaxant medicine with nitre and a little volatile alkali, and found, in the evening, figns of expectoration coming on, and which medicine now was confidered competent for the treatment of this fupervening difeafed affection.

At this time the breaft was not an object of attention, only the ordinary dreffing and cleanlinefs were attended to; and when it again became neceffary to be examined into, the ulcer was changed into a common fore, the breaft fhrunk, and confiderably abforbed; and in a fhort time the whole was taken up as well as the fwelling in the axilla, leaving for fome little time after, a fmall difcharge from the moft deep-feated ulceration, which alfo, in defiance of a ftimulating application to keep up a difcharge, clofed.

Ever fince her breath has been fhort, and checked with cough, and glary expectoration; the pulfe, which before, I believe, never exceeded 80, was now upwards of 130, creeping and indiftinct. She appeared exfanguinated, and very feeble; an iffue was made in the oppofite arm, but with no marked advantage.

On the 2d of October her oppreffive breath in fome degree increafed, with teafing cough, for which fhe was ordered her former mixture, with the addition of the tincture of Digitalis, which moderated both; but from my enforcing the neceffity of increafing the expectoration, fhe was afraid her medicine was mercurial, and did not purfue it properly.

Her breath and cough continuing troublefome, on the 21ft anafarcous fwelling of the face, ftuffing, and fullnefs in the bowels, came on, with a fcarcity of urine, fometimes high coloured, and with copious fediment; fometimes the urina jumentofa fecreting not more than four or fix ounces in twenty-four hours. In addition to her above mixture, fhe took ℥iv. of the cream of tartar in an electuary, with fome oil of anifeed, daily.

On the 24th the urine was a little increafed; and afraid of her her naufeating the medicine, I compreffed them into one dofe three times a day, or occafionally, as,

℞. Pulv. Digital. gr. j. Cryftal. tart. ʒij. Ipecac. gr. jß. Zinzib. gr. ij. Mifce.

Under this plan, the fecretion of urine was increafed; her pulfe, before hardly perceptible, became ftronger; her cough lefs troublefome; expectoration eafier, and more free, amounting to near a pint of glary mucus, fome a little yellow, in twenty-four hours.

On the fubfidence of the continued irritation in her breaft, and no appropriate fuccedaneum being made, I introduced a feton into the fide, ordering, over and above the powder, an infufion of cafcarilla with parfley-feed, changed afterwards for quaffia and carraway-feeds.

The pulfe being now 110, the fecretion of urine plentiful, a little rednefs of health appeared again in the lips, and the pulmonic affection being the principal object, I give her the following pill three times a day, taking a dofe of cream of tartar occafionally, with the infufion, conftantly three times a day.

℞. Pulv. Digital. gr. j. Ipecac. gr. jß. Ext. papav. alb. g. ß. Ol. anifi. g. jß. c. fyrup. fiat pilula.

With this the breath was expanded, and a purulent expectoration enfued; notwithftanding, much general relief was not felt till the 13th inftant; in the night a moft fevere pain attacked the right temple, and over the eye, which, in the morning, terminated in an eryfipelatous fwelling.

On the 15th the fwelling increafed, and blindnefs enfued; the breath and cough were much improved, from, probably, the difeafed irritation being determined to the face; this induced me to prefcribe patience only; and on the 18th it totally difappeared, but with an evident aggravation of the complaint in the cheft.

She now occafionally drinks to her meals, malt infufion in the different ftages of fermentation, with yeaft, in addition to her other plan.

In the reduced ftate Mrs. Craib was in, when the pleurify took place, perhaps much of its violence might have been owing to a mercurial habit, but not a little to about a pint of Madeira wine fhe drank from the commencement of it. The bleeding and relaxants employed, produced a quick abforption, and the general inflammation changed the ulceration to a common fore, which had fuch a tendency to heal, that it clofed up in defiance of a ftimulating ointment applied to keep it difcharging.

I have confined myfelf to the ftatement of facts; and how far art had to do with the changes that took place, I leave the profeffional gentlemen to determine. What I have now to regret is, that neither of them holds out hopes of remedy to fuffering humanity afflicted with fuch a dreadful difeafe as cancer; and though I admit of the removal of the difeafed breaft, and fwelling in the axilla, by an effort of the conftitution,* it is my opinion that neither of the cafes was cancer, certainly not idiopathic cancer. All who faw Mrs. Ofborn's cafe, faid it was a true cancer; but Mr. Lewis, furgeon, in Half Moon Street, who examined it at this time, was of a different opinion; he had his reafons; and the refult of the cafe fatisfies me his decifion was judicious. Mrs. Craib's was more equivocal.

* See the influence of febrile action in the Aphorifms of Hippocrates, "Febris morbum tollit." and Gaubius, "Licet enim commotio ifto, perturbato circulationis fyftemate gravibufque fymptomatis molefta, haud raro perniciem inferat, hinc merito morbus appelletur; fæpe tamen et mirifice adeo falutaris eft, ut certius aliud potentiusve cum ad fanandos, tum præcavendos morbus auxilium natura vel ars, vix agnofcat."

